# A not so incidental ‘incidentaloma’ − pediatric ganglioneuroma-associated cerebellar degeneration and super-refractory status epilepticus: case report and literature review

**DOI:** 10.3389/fneur.2023.1250261

**Published:** 2023-10-19

**Authors:** Albert Aboseif, Kaitlyn Palmer, Aaron W. Abrams, Deepak Lachhwani, Elia Margarita Pestana Knight, Ahsan Moosa Naduvil Valappil, Andrew Zeft

**Affiliations:** ^1^Department of Neurology, Cleveland Clinic, Cleveland, OH, United States; ^2^Mellen Center for Multiple Sclerosis, Cleveland Clinic, Cleveland, OH, United States; ^3^Epilepsy Center, Cleveland Clinic, Cleveland, OH, United States; ^4^Center for Pediatric Rheumatology, Cleveland Clinic Children’s Hospital, Cleveland, OH, United States

**Keywords:** paraneoplastic neurologic syndrome, autoimmune encephalitis (AE), ganglioneuroma, incidentaloma adrenal tumor, cerebellar degeneration, super-refractory status epilepticus (SRSE)

## Abstract

Paraneoplastic neurological disorders are rare in children, with paraneoplastic cerebellar degeneration (PCD) considered highly atypical. We describe a 13-year-old girl with progressive neurobehavioral regression, cerebellar ataxia, and intractable epilepsy presenting in super-refractory status epilepticus. After an extensive evaluation, her clinical picture was suggestive of probable autoimmune encephalitis (AE). Further diagnostic testing revealed a molecularly undefined neural-restricted autoantibody in both serum and CSF, raising suspicion over an adrenal mass previously considered incidental. Surgical resection led to a robust clinical improvement, and pathology revealed a benign ganglioneuroma. This report widens the spectrum of paraneoplastic manifestations of ganglioneuroma, reviews the diagnostic approach to antibody-negative pediatric AE, and raises important clinical considerations regarding benign and incidentally found tumors in the setting of a suspected paraneoplastic neurologic syndrome.

## Introduction

Convulsive status epilepticus (SE) is the most common neurological emergency in children (1). Super-refractory status epilepticus (SRSE) is defined as refractory status epilepticus >24 h despite general anesthesia or recurring with its withdrawal (2). The annual incidence of pediatric SE is 17–23 per 100,000, with SRSE accounting for 12–26% of cases (3, 4). In up to 60% of pediatric refractory SE, an underlying cause is never found (5). Acute symptomatic seizure is the most commonly identified cause of SRSE, with encephalitis (infectious, para-infectious, or autoimmune) making up approximately 40% of cases (6).

Seronegative autoimmune encephalitis (AE) causing SRSE is often challenging, as it requires prompt recognition and early aggressive treatment. We describe a 22-year-old female who presented at age 13 with a unique syndrome of neurobehavioral regression, progressive cerebellar degeneration, and seizures, eventually culminating in SRSE. This clinical phenotype and associated malignancy has not been previously reported. We review the literature on the approach to pediatric seronegative AE, the spectrum of paraneoplastic neurologic syndromes in childhood, and the neurologic associations of ganglioneuroma.

## Case report

A 22-year-old right-handed female with progressive cognitive decline and incoordination presented to our hospital at age 13 in SRSE. Born full-term without perinatal complications, she developed subacute cognitive and social regression at age 5, including short-term memory impairment, introversion, and social anxiety. At age 7 she developed episodic stomach pain and photopsia associated with spitting and repetitive speech. She concurrently demonstrated motor and language regression. Her examination revealed dysarthria, bilateral horizontal nystagmus, and limb and trunk ataxia impairing independent ambulation. She experienced anxiety and obsessive-compulsive disorder (OCD) resulting in germ-related obsessions and compulsive handwashing.

**Figure 1:**
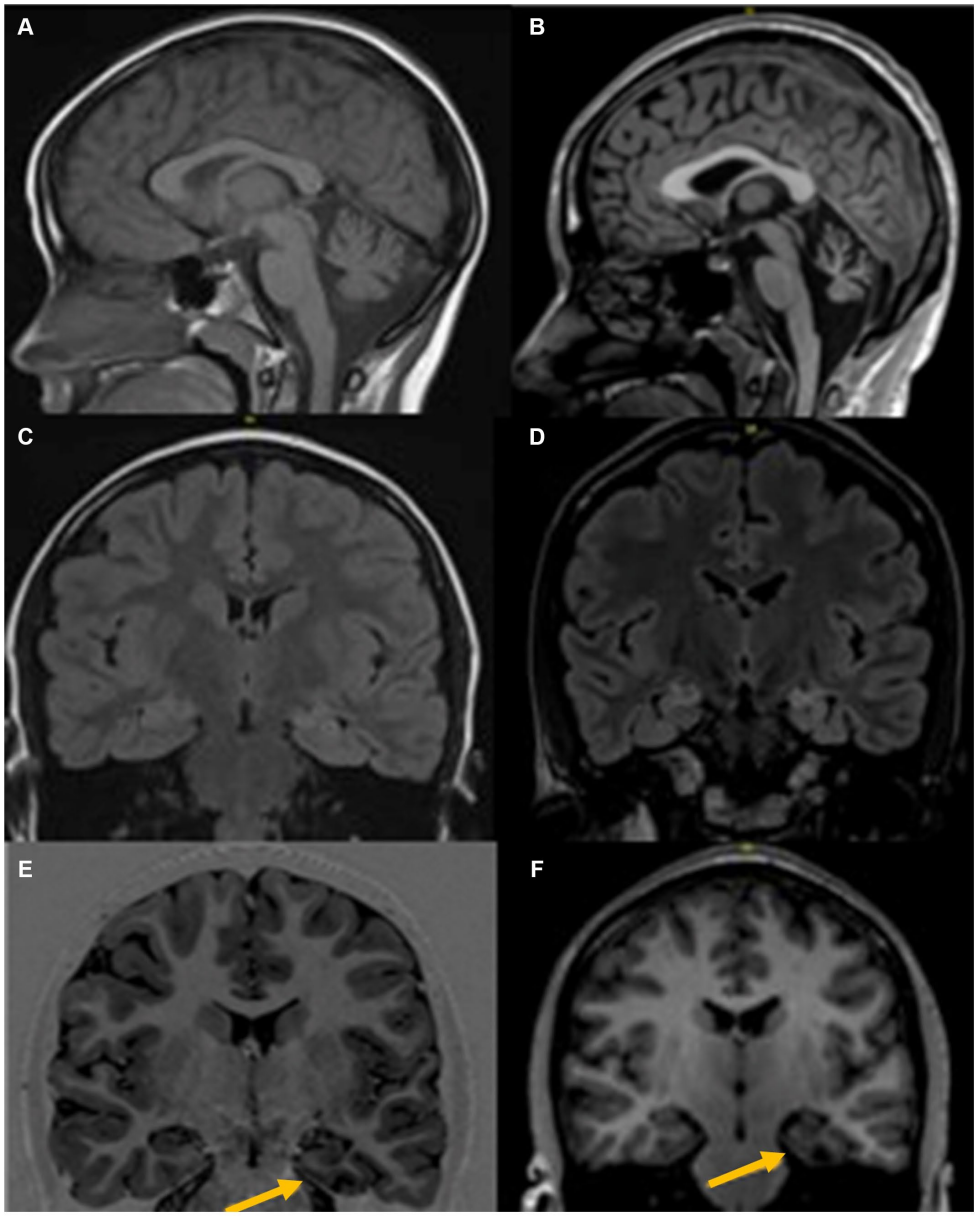
MRI Brain at age 6 years (left) and age 18 years (right), shows progressive changes with prominent diffuse cerebellar atrophy **(A,B)**, Signal change in both mesial temporal regions with left predominant atrophy **(C,D)**, and left parahippocampal atrophy **(E,F)**.

At age 8 she developed her first generalized tonic–clonic seizure (GTC) in addition to recurrent focal seizures characterized by ictal fear and asymmetric tonic posturing, leading to a diagnosis of multifocal epilepsy (Table 1). Between the ages of 8–11, she underwent extensive evaluations including metabolic, genetic, muscle, and skin biopsy, which were unremarkable (Table 1). Brain magnetic resonance imaging (MRI) showed cerebellar and left posterior parahippocampal atrophy, with bilateral mesial temporal lobe T2 hyperintensities (Figure 1). Cerebrospinal fluid (CSF) evaluations revealed presence of CSF-specific oligoclonal bands (OCB) and elevated neopterin at 45 and 66 nmol/L (normal 8–33 nmol/L). Serum and CSF paraneoplastic autoantibody panels (Mayo Clinic Laboratories, MN United States) were negative including AGNA-1, amphiphysin, ANNA-1, ANNA-2, ANNA-3, PCA-1, PCA-2, PCA-Tr, N and P/Q-type calcium channel, AChR ganglionic neuronal antibody and VGKC. Given a concern for autoimmune epilepsy, she received a combination of intravenous immunoglobulin (IVIG), steroids, mycophenolate mofetil, plasmapheresis (PLEX), and ketogenic diet, with minimal improvement in her symptoms. A screening kidney ultrasound revealed an incidental left adrenal mass determined to be hormonally inactive (Table 1), and serial imaging follow-up was planned. At age 11, she was cognitively performing 2–3 years below the age-expected mean and attending school with support services.

**Table 1:** Summary of extensive unremarkable evaluation completed and deemed non-contributory to diagnosis.

Metabolic	Genetic	CSF	Other serum	Biopsy	EEG
Carnitine plasma Carbohydrate Deficient Transferrin for Congenital Disorders of Glycosylation Amino acids, plasma and urine Porphyria screen, urine Sterol profile Peroxisomal panel Guanidinoacetate/ creatinine Mucopolysaccharidosis, urine Purine/pyrimidine, urine Organic acid, urine	Karyotype, leukocytes: 46XX, 425 bands Oligoarray, Mayo Polymerase gamma-1 (polG1) Mitochondrial DNA genome sequencing Whole exome sequencing Comprehensive epilepsy panel	Amino acids Pyridoxal phosphate 3-O-methyldopa Pyruvate Lactate IgG index Succinyladenosine 5-methyltetrahydrofoloate N-methyl-D-aspartate antibody Paraneoplastic panel (Mayo) 5-hydroxyindoleacetic acid Homovanillic acid Tetrahydrobiopterin	Cardiolipin antibody Glutamate decarboxylase antibody Anti-microsomal antibody Antinuclear antibody Estradiol Androstenedione Dehydroepiandrosterone sulfate Testosterone HCG Metanephrines Alpha-fetoprotein	Muscle histology for electron transport chain Skin tissue analysis with electron microscopy and fibroblast culture studies	EEG ages 7–13: Multifocal interictal spikes, polyspikes right frontal-central-temporal and left occipital Pre-surgical EEG (age 13): Right frontal-central status epilepticus Post-surgical EEG (age 13): Brief bursts of multifocal polyspikes and 2–9 brief tonic seizures daily EEG ages 14–19: interictal multifocal spikes intermittently captured on ambulatory EEG

**Figure 2:**
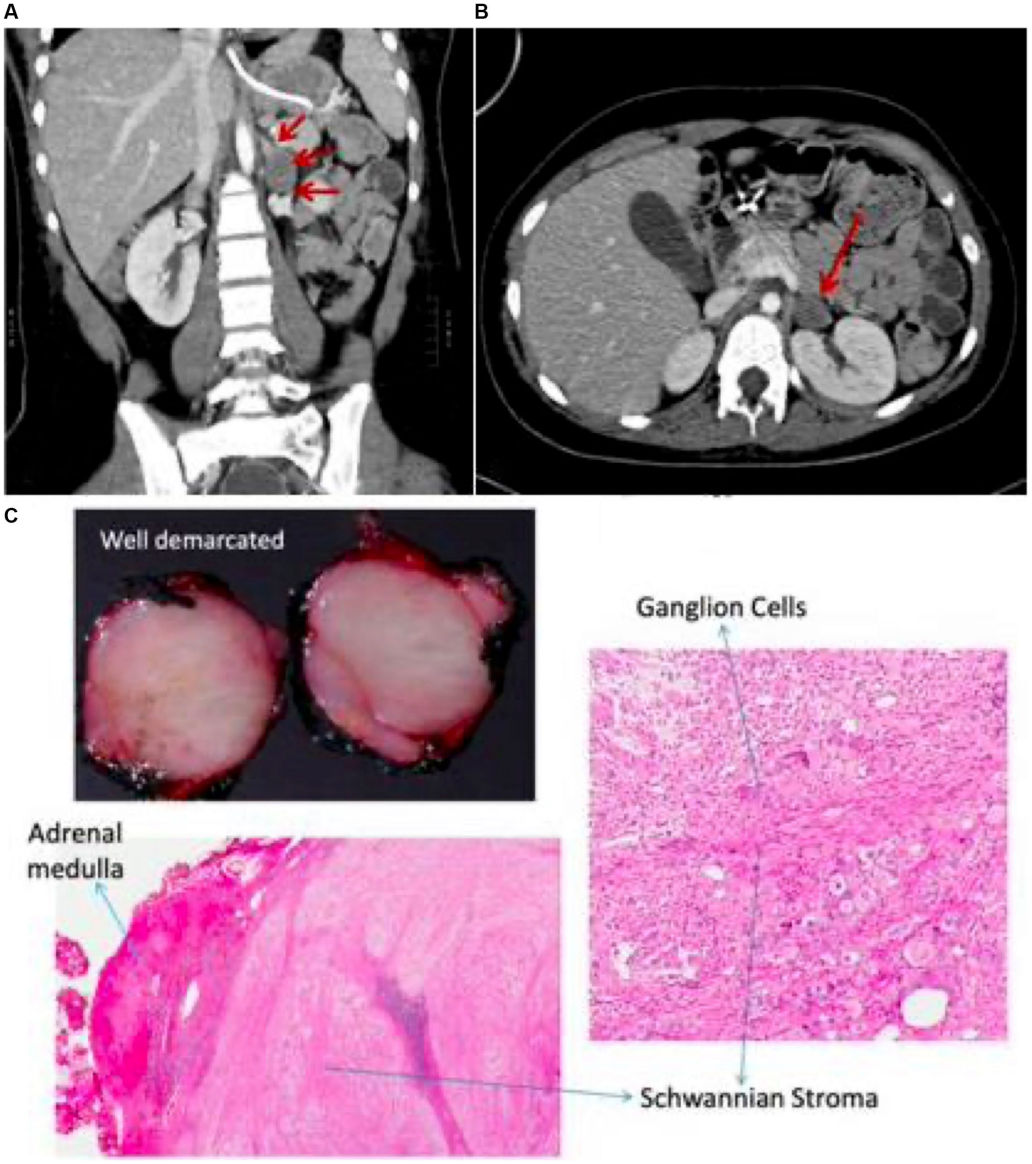
CT chest & abdomen shows non-enhancing mass in the left adrenal region **(A,B)** with corresponding histopathology demonstrating features of ganglioneuroma **(C)**.

**Figure 3:**
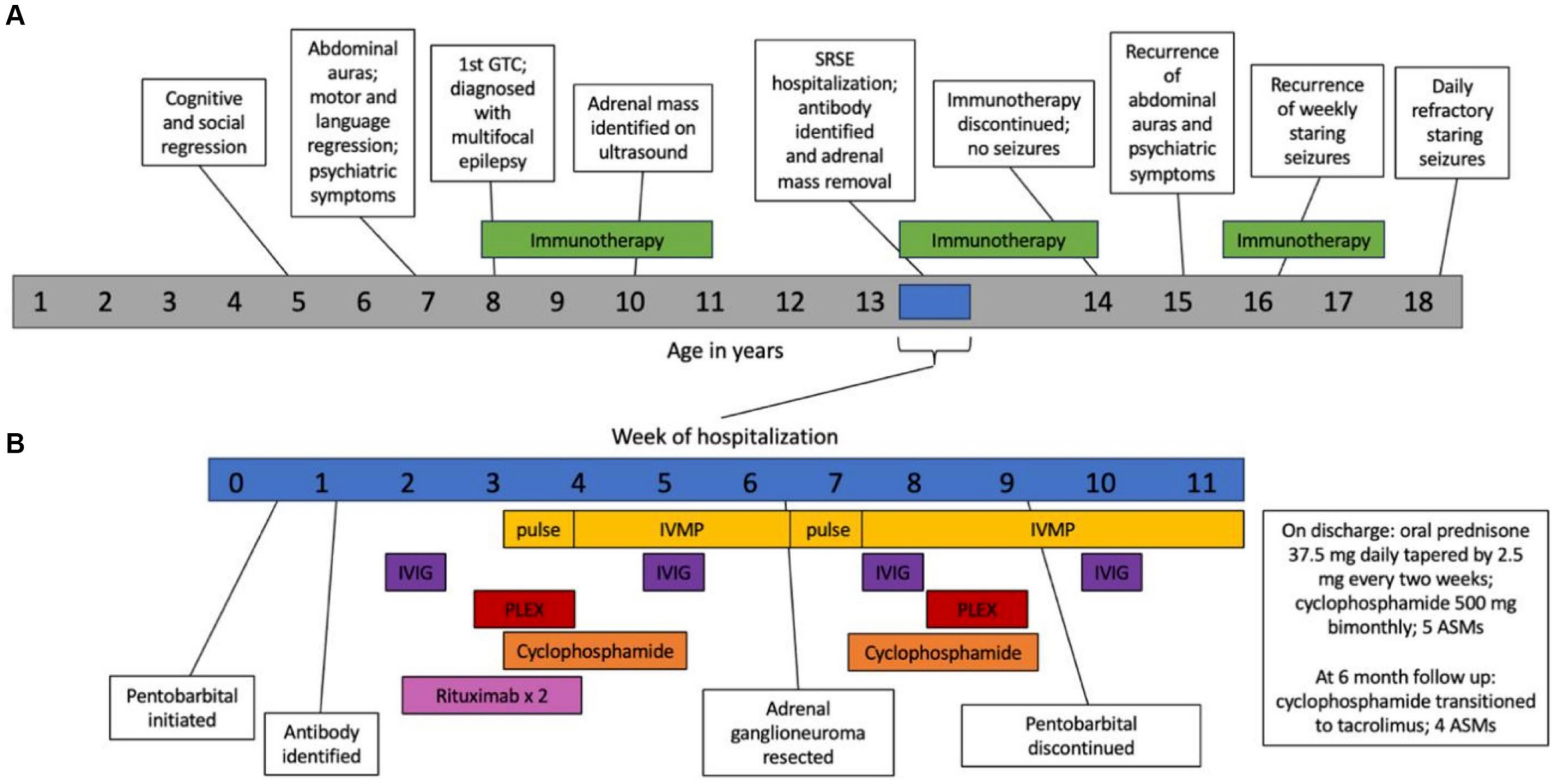
Timeline outlining disease progression and response to treatment over the lifespan **(A)** and during hospitalization for SRSE **(B)**. SRSE, super-refractory status epilepticus; GTC, generalized tonic clonic seizure; IVMP, intravenous methylprednisolone; PLEX, plasmapheresis; IVIG, intravenous immunoglobulin; ASM, anti-seizure medication.

At age 13 she developed increased seizure frequency leading to focal SRSE resulting in admission to our pediatric intensive care unit. Burst suppression was achieved with pentobarbital, but multiple efforts to wean sedation throughout her hospitalization led to refractory seizure activity. Repeat CSF testing showed lymphocytic pleocytosis (6 cells/uL), elevated protein (65 mg/L), IgG index of 0.60 (0.0–0.61), CSF-specific OCBs, and an elevated neopterin level (71 nmol/L). Serum and CSF paraneoplastic autoantibody panels (Mayo Clinic Laboratories, MN United States) were again negative. However, on indirect immunofluorescence of mouse tissue substrate, a yet molecularly undefined neural-restricted antibody was identified in both serum and CSF with strong binding affinity to purkinje-cells. On further review of her prior testing, the same immunofluorescence staining pattern was visualized on serum and CSF testing at ages 9 and 10. Due to a high suspicion for paraneoplastic AE, computed tomography (CT) scan of her abdomen was performed which re-demonstrated the left adrenal mass (Figure 2), and follow-up positron emission tomography (PET)/CT revealed corresponding hypermetabolism. Although her adrenal mass was hormonally inactive and stable in size from four years prior, a high suspicion for a paraneoplastic neurologic syndrome led to a multidisciplinary consensus to proceed with surgical resection. Due to continued seizure activity and associated hemodynamic instability, she first required preliminary treatment with three days of 1 g intravenous methylprednisolone (IVMP) followed by 40 mg of prednisone daily, one cycle of PLEX, two separate courses of 2 g/kg IVIG, two doses of 1 g rituximab, and six doses of 500 mg IV cyclophosphamide (Figure 3). Two months into her hospitalization, she finally underwent successful resection of her adrenal mass. Pathology was consistent with adrenal ganglioneuroma (Figure 2).

On post-op day one, she was able to begin weaning pentobarbital. She received an additional 1 g IVMP followed by 40 mg daily, one dose of cyclophosphamide, a second cycle of PLEX, and two additional courses of IVIG. Three weeks after surgery, pentobarbital was successfully discontinued after 63 days. Her seizures improved to episodes of brief facial grimacing occurring less than ten times per day on 5 anti-seizure medications (ASM). On discharge she was maintained on oral prednisone 37.5 mg daily with an extended taper of 2.5 mg every two weeks and 500 mg cyclophosphamide bimonthly. One month after tumor resection she was successfully discharged to a rehabilitation facility. Repeat paraneoplastic autoantibody testing in the serum was negative, and the previously visualized immunofluorescence staining pattern was no longer present. At six-month follow-up, she was walking independently with residual ataxia. Her anxiety and OCD improved, and she returned to school. Seizures were controlled on four ASMs. Cyclophosphamide was transitioned to tacrolimus. At age 14, 1 year after tumor resection, she was weaned off all immunotherapy and remained seizure-free for the next two years on four ASMs.

At age 15 she had increasing abdominal auras, along with recurrence of OCD and anxiety. At age 16 her seizures recurred. Repeat abdominal CT did not show tumor recurrence, serum immunofluorescence remained negative, and MRI brain was stable. She received IVMP with improvement in seizure frequency. At age 17 she began having staring seizures lasting 15–20 s occurring 1–2 times per week, increasing in frequency over the next several months. Immunosuppression with steroids, mycophenolate mofetil, and later tacrolimus was resumed with minimal improvement in seizure frequency. On follow-up at age 18, she had refractory daily staring seizures predominantly in the morning. Her exam showed bilateral gaze-evoked horizontal nystagmus, ataxic dysarthria, upper extremity ataxia, and wide-based ataxic gait. Given her non-inflammatory CSF profile, significant atrophy on MRI, and prolonged duration of symptoms, her worsening was felt to be secondary to chronic sequelae of her prior AE, with no active inflammation. Further immunosuppression was therefore not recommended, with symptomatic treatment pursued instead. At age 23, her mother reported only brief 30-s focal non-convulsive seizures occurring no more than three times daily. She did not require rescue medications, and her gait instability had stabilized with no further worsening since surgery.

## Discussion

The incidence of pediatric hospitalizations due to encephalitis in the U.S. between 2000–2010 was ~23 per 100,000 children, occurring predominantly in infants (7). No etiology is identified in 50% of cases, with viral infection the most commonly identified cause (8). AE is increasingly recognized among children, with anti-N-methyl-aspartate receptor (NMDAR) AE the most common cause (8, 9).

Unclassified autoantibodies are thought to be a potential cause of AE in a subset of pediatric cases (9). These patients often meet criteria for probable antibody-negative AE and may benefit from early immunotherapy (9). It is therefore important for clinicians to make a diagnosis of AE utilizing clinical and paraclinical data, without relying purely on confirmatory autoantibody testing. A set of diagnostic criteria specific to pediatric AE was recently proposed (9, 10). The criteria included the onset of neurologic and/or psychiatric symptoms over <3 months in a previously healthy child, two or more neurological symptoms (focal neurologic deficit, cognitive dysfunction or developmental regression, seizure, movement disorder, and/or psychiatric symptoms), at least one marker of neuroinflammation (CSF leukocytosis >5 cells/mm^3^, positive OCBs, MRI abnormalities) and reasonable exclusion of alternative etiologies (9).

Cases of antibody-negative AE are exceedingly challenging and require a high index of suspicion. In our patient for whom standard autoantibody testing was negative, utilization of the AE diagnostic criteria was critical, leading to tumor discovery and treatment response. Our patient’s progressive neurological syndrome, coupled with her inflammatory CSF profile, abnormal brain MRI, and neural-specific staining pattern on indirect immunofluorescence, raised increased suspicion for a possible paraneoplastic neurologic syndrome. Her adrenal mass had previously been ignored given its benign appearance and hormonal inactivity. However, a high suspicion for AE led to resection of her mass, and administration of immunotherapy perioperatively. Pathology ultimately revealed a neurogenic-derived tumor. Her ability to tolerate a reduction in intravenous anesthetic agents within days of tumor resection, after multiple failed attempts, further supported the diagnosis.

Although her clinical progression was chronic, she met the remaining clinical criteria, and underwent extensive diagnostic testing to exclude alternative etiologies including genetic and metabolic causes. While AE classically presents within 3 months, if the diagnosis is missed, patients can develop chronic disabling symptoms which are often difficult to treat, as depicted in this case (11, 12). Had her tumor been resected earlier in her disease course, she may have had a better overall outcome. Additionally, while a comorbid idiopathic childhood developmental disorder cannot be excluded, her improvement in neurobehavioral symptoms following treatment suggests they were likely an early manifestation of her paraneoplastic syndrome. Furthermore, while the immunotherapy administered during her hospital course likely contributed to her clinical improvement in combination with tumor removal, it is unlikely to have worked in isolation. This is evidenced by her lack of response to multiple immunotherapies at age 8 with continued clinical progression, coupled with her ability to wean anesthesia within days of tumor resection, after multiple failed attempts. Finally, while the progression of her seizure disorder in late adolescence raised concern for recurrence of her paraneoplastic syndrome, her CSF was non-inflammatory, and her abdominal imaging was negative. Paraneoplastic neurologic syndromes can often be progressive, even despite aggressive treatment (13, 14). Seizures can be refractory, and cerebellar ataxia is often residual (13, 14). Nonetheless, it remains important to maintain a high degree of suspicion for tumor recurrence with continued cancer surveillance.

Her initial clinical presentation was consistent with paraneoplastic cerebellar degeneration (PCD), a syndrome which is rare in children (15). A PubMed-based literature review using key words “paraneoplastic” and “cerebellar degeneration” or “cerebellar syndrome” and “pediatric” revealed only 10 reported cases of PCD over the last 30 years. Six involved children with Hodgkin’s lymphoma, four of whom had a positive Anti-Tr antibody, one with an unspecified purkinje-cell antibody, and one without an identified neural-specific antibody. The four other cases involved a PCA-1 antibody associated with adrenal adenocarcinoma, and a P/Q type calcium channel antibody without a known malignancy, with two additional cases having an underlying ganglioneuroma. Notably, most cases presented with pure cerebellar ataxia, and none were associated with refractory seizure activity.

Ganglioneuromas are benign neuroblastic tumors arising from sympathetic ganglion cells, with neuroblastoma representing the malignant form of the tumor (16). Ganglioneuromas are commonly asymptomatic, but can cause local compression, in addition to gastrointestinal and hemodynamic effects secondary to neuroendocrine peptide secretion (17). Unlike neuroblastomas, ganglioneuromas are rarely associated with a paraneoplastic neurologic syndrome. Prior reports have described an associated opsoclonus-myoclonus syndrome, and an ANNA-1 associated PCD (18, 19). Two additional cases showed a close resemblance with our case, including an 8-year-old girl with multifocal subcortical white matter lesions and cerebellar atrophy in the setting of a mediastinal ganglioneuroma, and a 4-year-old boy with chronic ataxia syndrome that improved following resection of an adrenal ganglioneuroma (20, 21). Only three additional pediatric cases in the literature have identified a neurologic syndrome associated specifically with adrenal ganglioneuroma – a 34-month-old child with ataxia-opsoclonus-myoclonus syndrome, a 7-year-old female with a seizure disorder, and a 2 ½ year-old female with cerebellar ataxia and opsoclonus (22–24). None reported an associated neural-restricted antibody (20–22).

In general, reports of pediatric neurological paraneoplastic syndromes are rare (23). The most common are opsoclonus-myoclonus syndrome which is often antibody-negative and associated with neuroblastoma (50%), NMDAR AE or classic limbic encephalitis secondary to anti-NMDAR-IgG with an underlying ovarian teratoma (at much lower rates than adults), anti-Ma2 (di)encephalitis classically associated with testicular germ cell tumors, and anti-acetylcholine receptor positive myasthenia gravis associated with thymic hyperplasia or thymoma (more often in adults) (25).

A combination of cerebellar ataxia and seizure has been described in a subset of autoantibodies including anti-SEZ6L2 with associated extrapyramidal features, anti-Ma-2, anti-ANNA-1, anti-glycine, anti-amphiphysin, and anti-DPPX in the setting of limbic encephalitis, anti-GAD-65 neurological autoimmunity, and in anti-kelch-like protein-11 with rhombencephalitis, among others (26–28).

## Conclusion

We present a case of an adrenal ganglioneuroma causing a progressive syndrome of neurobehavioral regression, paraneoplastic cerebellar degeneration, focal epilepsy, and SRSE. A yet molecularly uncharacterized autoantibody in serum and CSF was identified on tissue indirect immunofluorescence assay with predominant purkinje-cell binding, which may have important implications for future research. Our case underscores the importance of early recognition and treatment of clinical autoimmune encephalitis in children, even in the absence of seropositivity, as an unknown proportion of cases may have an as-yet-unidentified autoantibody-associated syndrome. Prompt tumor identification, resection, and consideration of additional immunotherapy should be explored. Incidentally found and benign appearing tumors should be closely evaluated and considered for resection if suspicion for a paraneoplastic neurologic syndrome is high. As the field of neuroimmunology continues to expand, it will be important to continue to report and define novel antibody-associated syndromes to assist in the pursuit of novel antibody discovery and targeted treatment.

## Data availability statement

The original contributions presented in the study are included in the article/supplementary material, further inquiries can be directed to the corresponding author.

## Ethics statement

Ethical review and approval was not required for the study on human participants in accordance with the local legislation and institutional requirements. Written informed consent from the patients/participants or patients/participants' legal guardian/next of kin was not required to participate in this study in accordance with the national legislation and the institutional requirements. Written informed consent was obtained from the minor(s)' legal guardian/next of kin for the publication of any potentially identifiable images or data included in this article.

## Author contributions

AlA: conceptualization, writing – original draft, writing – review and editing, visualization, and project administration. KP: writing – review and editing and visualization. AaA: writing – review and editing. DL: writing – review and editing. EK: conceptualization, writing- review and editing, and project supervision. AV: writing – review and editing and supervision. AZ: conceptualization, writing – review and editing, supervision, and project administration. All authors contributed to the article and approved the submitted version.
